# Simultaneous Detection of Different MicroRNA Types Using the ZIP-Code Array System

**DOI:** 10.1155/2013/496425

**Published:** 2013-09-02

**Authors:** Sonja U. Weishaupt, Steffen Rupp, Karin Lemuth

**Affiliations:** ^1^Institute of Interfacial Process Engineering and Plasma Technology (IGVP), University of Stuttgart, Nobelstraße 12, 70569 Stuttgart, Germany; ^2^Fraunhofer Institute for Interfacial Engineering and Biotechnology (IGB), Nobelstraße 12, 70569 Stuttgart, Germany

## Abstract

MicroRNAs (miRNAs) are important negative regulators of gene expression. Their implication in tumorigenesis is based on their dysregulation in many human cancer diseases. Interestingly, in tumor cells, an altered ratio of precursor and mature miRNA levels has been described. Consequently, differences in miRNA type levels have a high potential as biomarkers and comparative high-throughput-based detection might permit a more accurate characterization of subtypes, especially in the case of very heterogeneous tumor entities. Several molecular methods exist for the detection of mature and precursor miRNAs. DNA microarrays are predestinated as a high-throughput method for comprehensive miRNA detection in tumors. However, the simultaneous array-based detection of both these miRNA types is limited because the mature miRNA sequence is identically present in both forms. Here we present a ZIP-code DNA microarray-based system in combination with a novel labeling approach, which enables the simultaneous detection of precursor and mature miRNAs in one single experiment. Using synthetic miRNA templates, we demonstrate the specificity of the method for the different miRNA types, as well as the detection range up to four orders of magnitude. Moreover, mature and precursor miRNAs were detected and validated in human tumor cells.

## 1. Introduction

MicroRNAs (miRNAs) are small noncoding RNAs that are known to have important regulatory functions in gene expression and influence various biological processes—like cell growth, differentiation, and apoptosis in eukaryotes (reviewed in [1, 2]). Because of their involvement in these basic cellular processes, miRNAs also play an important role in tumor development (reviewed in [3–5]). Genes encoding miRNAs are located on the chromosome as independent transcription units, separately from previously annotated genes, either within introns or even within exons [6]. Some miRNA genes are clustered and transcribed as multicistronic primary transcripts. miRNAs in these transcription units are often but not always related to each other. Further, not all miRNAs of the same cluster are active at the same time [7]. 

miRNA biogenesis starts from an up to several kilo bases long primary miRNA transcript (pri-miRNA) in the nucleus that contains a hairpin structure from which the mature miRNA is processed. Pri-miRNAs are with a few exceptions transcribed by RNA polymerase II [8, 9]. A complex including the RNase III endonuclease Drosha and the double-stranded RNA binding domain protein DGCR8 further processes them to short hairpin precursor miRNAs (pre-miRNA), 60–110 nucleotides (nt) in length [10, 11]. Pre-miRNAs are exported by Exportin-5 in a Ran-GTP-dependent mechanism to the cytosol [12] where they are finally processed by a complex containing Dicer, another RNase III enzyme, and TRBP/Loquacious to yield the regulatory active 19–24 nt long mature miRNAs in the cytosol [13–15]. Mature miRNAs are incorporated into RNA-induced silencing-complex- (RISC-) like ribonucleoprotein particles (miRNPs) and target specific mRNAs, to trigger mRNA degradation, translational repression, or both [6, 16, 17].

miRNA processing is regulated in development, differentiation, and cancer (reviewed in [18]). Changed levels of mature miRNAs but unchanged levels of pri- and pre-miRNAs are the hallmark of regulated miRNA processing. Posttranscriptional regulation of miRNA processing has been described on the Drosha and Dicer levels [19–21].

Interestingly, in tumor cells, for example, in lung cancer [3, 22] or primary effusion lymphoma [23], an altered ratio of precursor and mature miRNA levels has been described. The profiling of different miRNA types in parallel is important for a comprehensive cancer classification [23], especially in the case of very heterogeneous cancers—like lymphomas [24]. Consequently, differences in miRNA type levels have a high potential as biomarkers and their detection might allow a more accurate characterization of different tumor subtypes.

Currently, precursor and mature miRNAs are detected by several molecular methods (e.g., northern-blot [25–27], qRT-PCR [28–30], and DNA microarrays [3, 31, 32]). Northern-blot analysis allows the simultaneous detection of all miRNA types in parallel. However, the method is laborious and limited in the number of analytes measured in parallel [33]. qRT-PCR offers a higher sensitivity for detection, but it also performs on low-throughput level [34]. 

In contrast, DNA microarrays are predestinated as a high-throughput method for miRNA detection in tumors. However, commercially available array systems are not able to detect the different miRNA types simultaneously without costly size exclusion (GeneChip miRNA 3.0 Array, Affymetrix, Santa Clara, USA) or approximate data analysis (GenoExplorer microRNA system, GenoSensor Corporation, Tempe, USA), respectively, because these array systems only detect the mature miRNA target sequence characteristic for both miRNA types. 

Here we show a ZIP-code DNA microarray-based system, similar to Hauser et al. [35] that allows the simultaneous detection of precursor and mature miRNAs in one single experiment. The array-platform setup uses unique and distinct ZIP-code probes that show no cross-hybridization to any known organisms. Specific primers attached to complementary ZIPs (cZIPs) are used for the specific labeling reaction. This allows the simultaneous detection of transcript level variations, genotypic differences, and DNA-protein interactions [35]. We combined this array platform using conventional DNA oligomers with a novel labeling approach to simultaneously detect precursor and mature miRNAs on a DNA microarray in one single experiment for a set of nine miRNAs. The approach has the potential for genome-wide miR analysis.

## 2. Material and Methods

### 2.1. Cell Lines and Chemicals

Human cervix carcinoma cell line (HeLa) was obtained from the German Collection of Microorganisms and Cell Cultures (Braunschweig, Germany). Keratinocyte cell line (HaCaT) was kindly provided by Professor Dr. Fusenig (DKFZ Heidelberg, Germany). 

All chemicals and reagents were obtained from Carl Roth (Karlsruhe, Germany) or as indicated, and they were of the highest available purity. 

### 2.2. Cultivation of Human Cell Lines

HeLa and HaCaT cell lines were cultivated in 75 cm^2^ cell culture flasks (Greiner, Frickenhausen, Germany) and split 1 : 3 by standard methods, just before reaching confluence.

All cell culture media were purchased from GIBCO (Life Technologies, Darmstadt, Germany). HeLa cells were maintained in RPMI 1640 and HaCaT cells in DMEM (high glucose), each supplemented with 10% fetal calf serum (FCS), 1% L-glutamine, and 1% Penicillin-Streptomycin.

All cells were cultivated under standard conditions at 37°C and 5% CO_2_ and passaged using Ca^2+^-Mg^2+^-free media for HeLa cells and 10x trypsin-EDTA for HaCaT cells.

### 2.3. Primers and Oligonucleotides

All primers and oligonucleotides used in this work were purchased from Metabion (Planegg, Germany). qPCR primers and T7 promoter oligonucleotides were ordered HPLC purified; ZIP-code oligonucleotides, synthetic oligonucleotides, and miRNA specific oligonucleotide-cZIPs were ordered desalted.

### 2.4. Distinct Labeling of Precursor and Mature miRNA

#### 2.4.1. Preparation of Synthetic miRNA Templates

Using the following procedure, synthetic miRNAs with a 5′-polyA-tail were prepared similar to a protocol for linear amplification of miRNAs published by Mattie et al. [36].

A set of 9 miRNAs known to be involved in tumorigenesis was selected, and corresponding mature and stem-loop sequences were extracted from miRBase database V.18 (http://www.mirbase.org/; corresponding miRBase accession numbers; see Table 1). Oligonucleotides reverse complementary (rc) to the corresponding miRNA sequences with a maximum length of 80 nucleotides were designed containing a 14-mer poly d(T) sequence and the rc T7 RNA polymerase promoter sequence at the 3′-end (Table 1).

From these miRNA DNA oligonucleotides, T7-promoter-linked DNA oligonucleotides were produced; therefore, 10 *μ*M of T7 promoter oligonucleotides (5′-TAATACGACTCACTATAGGG-3′) was mixed with 10 *μ*M of miRNA DNA oligonucleotides and denatured for 3 min at 95°C, and strands were annealed at room temperature over night. These T7-DNAs were used as an input template for *in vitro* transcription (IVT).

Individual IVT reactions were performed for each T7-DNA, all containing 5x transcription buffer (Fermentas, St. Leon-Rot, Germany), 2 mM NTPs (GE Healthcare, München, Germany), 50 U RNase inhibitor (RNase Out, Invitrogen, Darmstadt, Germany), 30 U T7 RNA polymerase (Fermentas, St. Leon-Rot, Germany), and 1 *μ*g of the individual T7-DNA. IVT reactions were incubated for 14 hours at 37°C, followed by purification of the amplified miRNA with RNeasy Min Elute Cleanup Kit (Qiagen, Hilden, Germany) according to the manufacturer's instructions. Quantification of RNA amplificates was performed spectrophotometrically (V-630, Jasco, Gross-Umstadt, Germany). 50 ng of each synthetic miRNA template was used for cDNA preparation and distinct fluorescent labeling (see Section 2.4.4 cDNA preparation and distinct fluorescent labeling).

#### 2.4.2. miRNA Isolation and Amplification from Human Cells

miRNA <200 nt from cell lines was isolated from 10^7^ cells using mirPremier microRNA Isolation Kit (Sigma-Aldrich, Taufkirchen, Germany) according to the manufacturer's instructions for small RNA isolation from mammalian cell cultures. Isolated miRNA was analyzed by capillary electrophoresis (Agilent Small RNA kit, 2100 Bioanalyzer, Agilent Technologies, Waldbronn, Germany), and RNA concentration and quality were determined photometrically (NanoDrop 2000c, Peqlab, Erlangen, Germany or V-630, Jasco, Gross-Umstadt, Germany). Only RNA with 260/280 nm ratios of 1.8 to 2.0 was used for reverse transcription and labeling.

Amplification of miRNA was performed using NCode miRNA Amplification System (Invitrogen, Darmstadt, Germany) according to the manufacturer's protocol, starting from 30 ng enriched miRNA. The *in vitro *transcription reactions were carried out for 16 h, followed by purification of the amplified 5′- and 3′-polyA-tailed miRNA molecules using RNeasy MinElute Cleanup Kit (Qiagen, Hilden, Germany) according to the manufacturer's protocol. 1.5 *μ*g of this amplified total miRNA was used for cDNA preparation and distinct fluorescent labeling (see Section 2.4.4 cDNA preparation and distinct fluorescent labeling).

RNA concentration and quality were determined photometrically (NanoDrop 2000c, Peqlab, Erlangen, Germany or V-630, Jasco, Gross-Umstadt, Germany). 

#### 2.4.3. miRNA-Specific cZIP-Code Design

Oligonucleotides reverse complementary (rc) to the corresponding mature miRNA sequences were extended 5′ with D-DNA cZIP sequences [35] (Table 2).

Each mature miRNA specific oligonucleotide (MSO) was linked to two different cZIP-code sequences; that is, two combinations of mature miRNA specific oligonucleotide-cZIPs (MSO-cZIP) for every miRNA were available.

#### 2.4.4. cDNA Preparation and Distinct Fluorescent Labeling

cDNA synthesis was performed via reverse transcription by incorporation of different fluorescence labeled deoxynucleotides (Cy3-ATP or Cy3-dUTP). 

As starting material for cDNA synthesis and distinct fluorescent labeling of synthetic miRNA, 50 ng synthetic templates of each miRNA were pooled separately for mature and precursor miRNAs. 

As starting material for cDNA synthesis and distinct fluorescent labeling of mature or precursor miRNA from cells, 1.5 *μ*g of amplified total-miRNA was used, respectively.

Labeling with Cy3-dUTP and Cy3-dATP was performed individually in two distinct reaction cups as follows: 0.25 pmol of each MSO-cZIP (either A or B; see also Table 2) was added to a volume of 10 *μ*L, followed by RNA denaturation for 5 min at 70°C and annealing of the MSO-cZIPs to their corresponding miRNAs for 30 min at 50°C.

For labeling with Cy3-dUTP, the annealed miRNA template was combined in a final 20 *μ*L reaction mix containing 5x reaction buffer (Fermentas, St. Leon-Rot, Germany), 0.5 mM dTTP (Qiagen, Hilden, Germany), 20 U RNase Out (Invitrogen, Darmstadt, Germany), 18.75 mM MgCl_2_ (Invitrogen, Darmstadt, Germany), 40 U M-MuLV Reverse Transcriptase (Fermentas, St. Leon-Rot, Germany), and 0.05 mM Cy3-dUTP (GE Healthcare, München, Germany).

For labeling with Cy3-dATP, 0.5 mM of each dCTP, dGTP, and dTTP and 0.08 mM dATP (Qiagen, Hilden, Germany) were used instead of 0.5 mM dTTP. Further, 0.05 mM Cy3-dATP (Perkin Elmer, Waltham, Massachusetts, USA) was used instead of 0.05 mM Cy3-dUTP.

The reverse transcription reaction was performed for 1.5 hours at 37°C and subsequently heat inactivated at 70°C for 10 min. After adding 2 U Ribonuclease H (Invitrogen, Darmstadt, Germany) to each sample to degrade RNA and incubation for 20 min at 37°C, samples were unified, followed by column purification of cDNA by QIAquick Nucleotide Removal Kit (Qiagen, Hilden, Germany). cDNA was eluted with 31.5 *μ*L elution buffer. 

### 2.5. Preparation of Genomic DNA

Genomic DNA was extracted from HaCaT cell pellets containing 10^7^ cells, using AllPrep DNA/RNA Mini Kit (Qiagen, Hilden, Germany) according to the protocol “*Genomic DNA Purification Protocol from Animal Cells*.” DNA concentration and quality were determined photometrically.

### 2.6. ZIP-Code DNA Microarrays

#### 2.6.1. Spotting

D-DNA ZIP-code oligonucleotides (Table 2) [35] with 3′ Amino-C7 linker (50 *μ*M in spotting buffer 1/3 (v/v) Nexterion Spot I/Spot III (Schott, Mainz, Germany)) were spotted in 16 replicates onto epoxy-coated glass slides (Nexterion Slide E, Schott, Mainz, Germany), using a MicroGrid II spotter (Digilab, Holliston, USA).

Slides were postprocessed and prehybridized according to the manufacturer's instructions.

#### 2.6.2. Hybridization, Scanning, and Data Analysis

13.5 *μ*L hybridization buffer (4x SSC and 0.1% SDS) was added to 31.5 *μ*L cDNA solution. Hybridization was performed using LifterSlips (Erie Scientific, Anaheim, USA) for 16 h at 65°C in a hybridization chamber. Slides were washed according to the manufacturer's instructions and scanned with an Axon GenePix 4300A scanner (Molecular Devices, Sunnyvale, USA) with a resolution of 10 *μ*m, laser power of 100%, and PMT values ranging from 500 to 800. Spot quantification was performed using GenePix Pro 7 software (Molecular Devices, Sunnyvale, USA). DNA microarray data analysis was based on averaged background-corrected median values obtained from GenePix Pro 7 software.

#### 2.6.3. Quantitative Real-Time PCR

To validate ZIP-code array results, quantitative real-time PCR (qRT-PCR) of mature and precursor miRNAs of HeLa cells was performed.

Isolated enriched miRNA was treated with Ambion TURBO DNase (Life Technologies, Darmstadt, Germany) to remove genomic DNA. Therefore, up to 500 ng small RNA was mixed in a final volume of 50 *μ*L with 10x Turbo Buffer and 0.6 U Turbo DNase I. After incubation at 37°C for 30 min, miRNA was purified using RNA Clean & Concentrator-5 (Zymo Research, Irvine, USA) according to the manufacturer's instructions. This kit allows the purification and concentration of RNA >17 nt.

100–500 ng of miRNA was reverse transcribed into cDNA via miScript II RT Kit (Qiagen, Hilden, Germany), according to the manufacturer's instructions using 5x miScript HiFlex Buffer, which enables conversion of mature as well as precursor miRNA.

**Figure 1:**
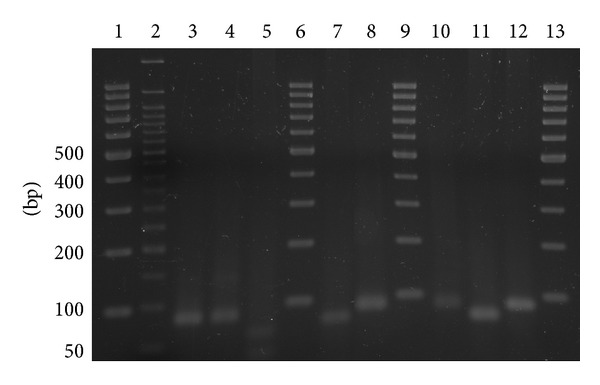
Agarose gel electrophoresis of qRT-PCR amplicons. qRT-PCR products were separated in a 3% agarose/1x TAE gel at 120 V for 1 h and stained with ethidium bromide. Lane 1: 100 bp marker (Fermentas, St. Leon-Roth, Germany); lane 2: 50 bp marker (NEB, Ipswich, USA); lane 3: miR-99a; lane 4: pre-miR-199b; lane 5: pre-miR-9; lane 6: 100 bp marker; lane 7: pre-miR-16; lane 8: miR-16; lane 9: 100 bp marker; lane 10: miR-9; lane 11: pre-miR-99a; lane 12: pre-miR-199b; and lane 13: 100 bp marker.

For qPCR, specific primers for individual miRNAs were used, either from commercial miScript Assays (Qiagen, Hilden, Germany) [37] or self-designed primers in a final concentration of 0.1 *μ*M (see Table 3). Self-designed primers were adjusted to a length of 20–25 nt and a melting temperature of 60°C ± 1°C using ApE software v1.17. Primers were designed and inspected with OligoAnalyzer 3.1 (IDT) to exclude self-dimers or oligo secondary structures. The specificity of primer pairs was checked using Basic Local Alignment Search Tool (BLAST; http://blast.ncbi.nlm.nih.gov/). Specificity of qPCR assays was additionally validated using gel electrophoresis (3% agarose (Carl Roth, Karlsruhe, Germany) and 1x TAE buffer (Tris acetate 40 mM/EDTA 1 mM, pH 8.0; each Carl Roth, Karlsruhe, Germany), 120 V, 1 h). Only the expected amplicon length (either mature or precursor) for each primer pair could be detected (see Figure 1). Further, the specificity of commercial assays has been confirmed by Qiagen.

The QuantiTect SYBR Green PCR Kit (Qiagen, Hilden, Germany) was used for qPCR, following protocols *Real-Time PCR for Detection of Mature miRNA or Noncoding RNA* and *Real-Time PCR for Detection of Precursor miRNA*, respectively.

qRT-PCR reactions were performed in Light Cycler 480 multiwell plates 96 in a volume of 25 *μ*L, with an input amount of 3 ng cDNA from reverse transcription reaction for detection of mature miRNA and 10 ng cDNA for precursors, respectively. All assays were performed at least in duplicates, and *no template-controls* (NTC) were included using water instead of cDNA as negative control. qPCR was performed on a LightCycler 480 Instrument (Roche, Mannheim, Germany) according to the following PCR protocol: 95°C for 15 min, 45 cycles of 94°C for 15 s, 55°C for 30 s, and 70°C for 30 s, followed by a melting curve analysis (65°C to 97°C) for determination of specificity of amplification. Further, specificity of qPCR products was validated using agarose gel electrophoresis (see Figure 1).

Standard curves for determination of the amplification efficiency of each assay were generated using a dilution series of cDNA from HeLa (in the range of 10^0^ and 10^−4^) or genomic DNA from HaCaT cells (2.4 × 10^5^–2.4 × 10^1^ copies/*μ*L), respectively. For PCR efficiency of individual primer pairs, please see Table 3.

## 3. Results and Discussion

We established a method for the simultaneous detection and distinction of mature and precursor miRNAs in one single experiment based on an established ZIP-code DNA microarray system with universal probes [35] and a novel labeling procedure. For this purpose, the miRNAs had to be prepared as follows.

Starting with an initial linear amplification of isolated miRNA [36], amplified miRNA (amiR) molecules with 5′- and 3′-polyA-tails were generated. On average 442 ng ± 150 ng amiR could be generated from 30 ng of isolated miRNA.

**Figure 2:**
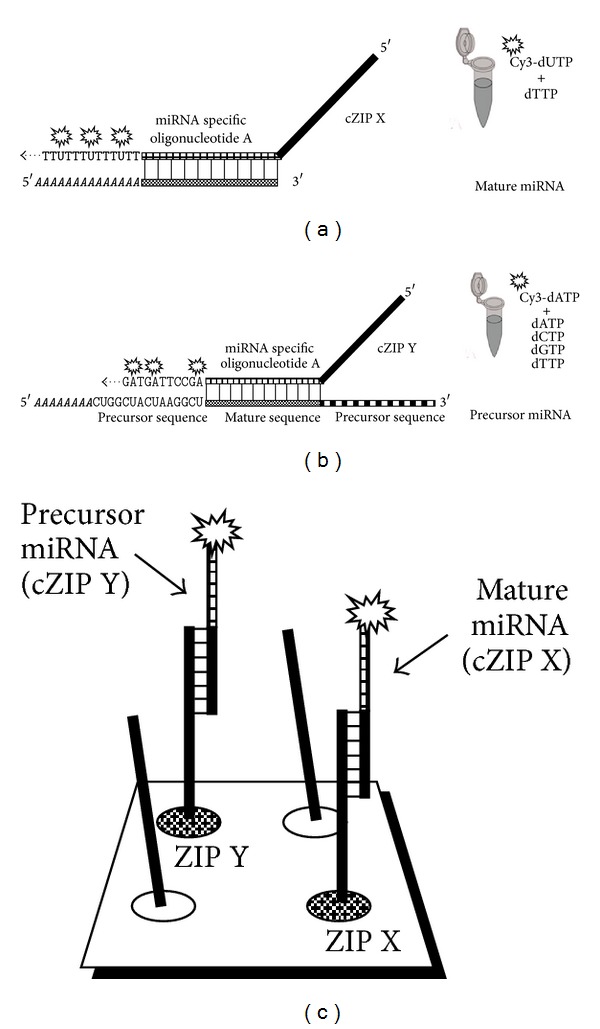
Labeling method for mature (a) and precursor (b) miRNA and their specific detection on ZIP-code array (c). Mature miRNA is labeled at the polyA-tail with Cy3-dUTP and unlabeled dTTP using miRNA-specific cZIP-Primer X; precursor miRNA is labeled at the consecutive precursor sequence with Cy3-dATP and unlabeled deoxynucleotides using miRNA-specific cZIP-Primer Y. The detection and differentiation of mature and precursor miRNA are based on the hybridization of the corresponding ZIP-code parts of the miRNA-specific cZIP-Primers to their complementary ZIP-code sequences immobilized at defined positions on the ZIP-code array.

The amiR 5′-polyA-tail of mature amiR was labeled by incorporation of Cy3-dUTP in an enzymatic reaction containing only Cy3-dUTP and unlabeled dTTP (Figure 2(a)). In this case, precursor amiR will not be labeled because of the subsequent bases following the mature sequence. However, one can get a false-positive signal if the first subsequent base is an A. In this case, the MSO should be extended by a 3′-T to avoid this.

In the case of precursor amiR, the precursor sequence following the MSO sequence was labeled using Cy3-dATP and all four unlabeled deoxynucleotides (Figure 2(b)) in an individual reaction. Accordingly, mature amiR cannot be labeled on its polyA-tail in this reaction. 

In the specific amiR labeling reactions, MSOs for each miRNA linked to two different cZIP-code sequences (MSO-cZIPs) were used. The ZIP-code part of the MSO-cZIPs allowed their distinction by the subsequent addressing to individual positions on the ZIP-code microarray (Figure 2(c)).

To ensure the comparability of the two distinct labeling approaches, we looked for differences caused by the ZIP-code pairs and differences caused by the different fluorescently labeled deoxynucleotides (Cy3-dATP, Cy3-dUTP). We tested ZIP-code pair-miRNA combinations for specificity as described in the next sections. 

Synthetic templates were used in combination with two different MSO-cZIP sets A and B, respectively (Table 2). All array experiments were performed in triplicates. ZIP-code pair-miRNA combinations were regarded as accurate when no significant differences within the standard deviations of the fluorescent signals could be detected. Additionally, no unspecific signals should occur on the DNA microarray. Only ZIP-code pair-miRNA combinations that pass these criteria are useful for distinction of mature and precursor miRNA. In that case, detectable differences on the DNA microarray are due to differences in miRNA type levels only.

The specificity of the ZIP-code array for a clear distinction of different miRNA types was examined by exclusive labeling of mature but not precursor synthetic miRNA and of precursor but not mature synthetic miRNA, respectively, and by competitive hybridization thereafter.

**Figure 3:**
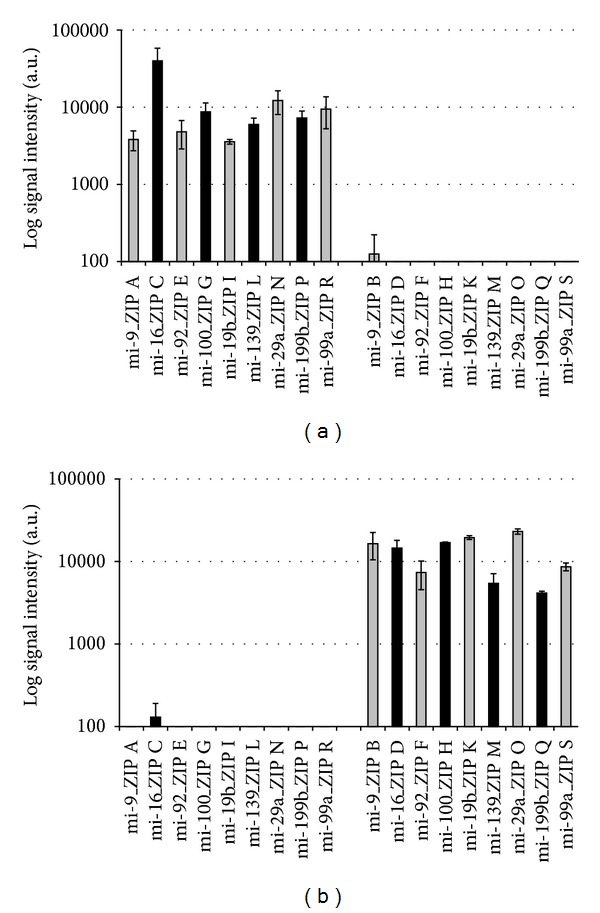
Exclusive detection of mature (a) and precursor (b) miRNA. Log signal intensities from three hybridizations of fluorescently labeled MSO-cZIPs with the ZIP-code array after specific labeling of synthetic miRNA templates are presented. (a) Specific labeling of mature synthetic miRNA templates with Cy3-dUTP and unlabeled dTTP using MSO-cZIP set A, and precursor synthetic miRNA templates were reverse transcribed with unlabeled deoxynucleotides and MSO-cZIP set B; (b) Specific labeling of precursor synthetic miRNA templates with Cy3-dATP and unlabeled deoxynucleotides using MSO-cZIP set B, and mature synthetic templates were reverse transcribed with unlabeled deoxynucleotides and MSO-cZIP set A. ZIPs for mature miRNA detection: A, C, E, G, I, L, N, P, and R; ZIPs for precursor miRNA detection: B, D, F, H, K, M, O, Q, and S.

To test for cross-hybridization in a first approach, labeling of mature synthetic miRNA was performed with Cy3-dUTP and unlabeled dTTP using MSO-cZIP set A, whereas labeling of precursor synthetic miRNA was performed exclusively with unlabeled deoxynucleotides and MSO-cZIP set B. The hybridization resulted in a specific detection of mature miRNA in 100% of all cases as shown in Figure 3(a). No cross-hybridization could be detected.

In a second approach, labeling of precursor synthetic miRNA was performed with Cy3-dATP and unlabeled deoxynucleotides using MSO-cZIP set B, and labeling of mature synthetic templates was performed exclusively with unlabeled deoxynucleotides and MSO-cZIP set A. This hybridization exhibited no significant signals for mature miRNA and confirms the specificity of the method for exclusive detection of precursor miRNA (see Figure 3(b)). 

The threshold for significant signals was set to 190 a.u., based on unspecific background signal intensity (averaged across three experiments) (see Figure 3(a)).

Additionally, specificity studies were performed using varying combinations of different synthetic mature and precursor miRNA templates. Labeling of mature miRNA with Cy3-dUTP and unlabeled dTTP using MSO-cZIP set A and precursor miRNA with Cy3-dATP and unlabeled deoxynucleotides using MSO-cZIP set B led to the specific pattern expected (data not shown). These results confirmed the specificity of the system for distinct detection of mature and precursor miRNAs.

The distinct detection of precursor and mature miRNAs using the ZIP-code array is based on the same miRNA sequence coupled to two different ZIP-code oligos and distinct labeling. To ensure that the different ZIP-code sequences of the appropriate ZIP-code pairs do not lead to significant signal differences, identical miRNA synthetic templates were labeled using corresponding miRNA-specific oligonucleotides in two different cZIP combinations.

**Figure 4:**
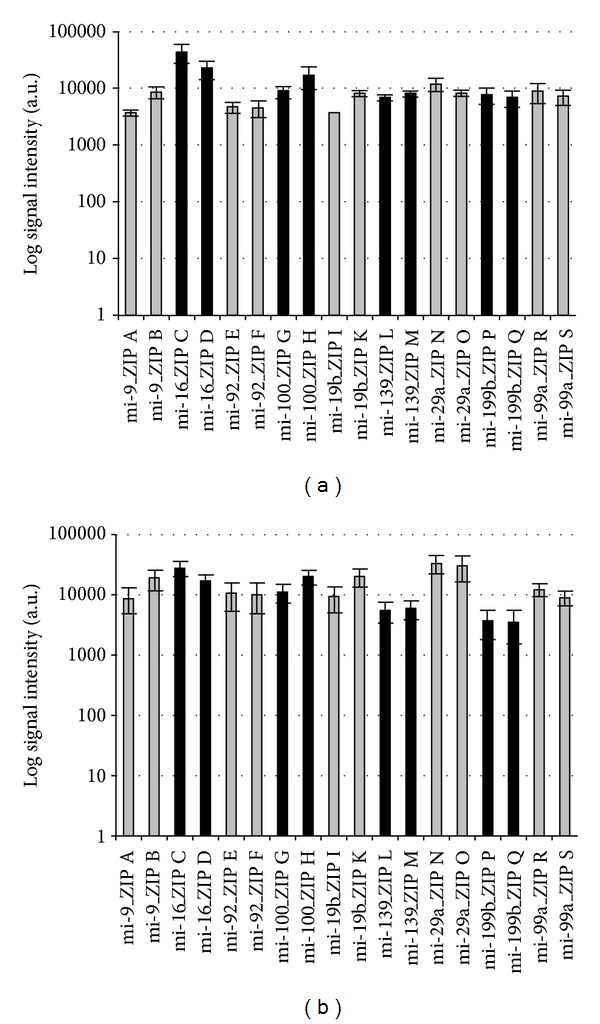
Comparison of different cZIPs linked to identical miRNA oligonucleotides based on synthetic mature (a) and precursor (b) miRNA template labeling. Using identical miRNA-specific oligonucleotides with different cZIPs in separate Eppendorf cups, mature synthetic miRNA templates were labeled with Cy3-dUTP and unlabeled dTTP (a), or precursor synthetic miRNA templates were labeled with Cy3-dATP and unlabeled deoxynucleotides (b). Log signal intensities from subsequent hybridization to the ZIP-code array in triplicates are shown. Different cZIPs for identical miRNA are illustrated contiguous in identical colors.

Mature miRNA synthetic templates were reverse transcribed with Cy3-dUTP and unlabeled dTTP using MSO-cZIP set A and MSO-cZIP set B, respectively. The experiments were performed in separate Eppendorf cups. After the simultaneous hybridization of both solutions to the ZIP-code array, signal intensities of corresponding ZIPs for each miRNA were compared (see also Figure 4(a)). Comparable signal intensities could be achieved for seven of the nine ZIP-code pairs. Only for ZIP-code pairs ZIP A/B and ZIP I/K differences in signal intensities beyond the standard deviation could be detected. However, the absolute signal intensities were in a comparable range, and therefore these ZIP-code pairs were also included in the following experiments.

The same experimental design was set up with precursor synthetic miRNA templates using Cy3-dATP and unlabeled deoxynucleotides for labeling (see also Figure 4(b)). In this experiment, except for ZIP pair I/K, comparable signal intensities could be detected in all cases.

The concordant results indicate that in the case of ZIP-code pair I/K variances might be based on variable ZIP binding efficiencies of the *in silico *designed D-DNA cZIPs. In these cases, respective ZIPs must be excluded from the microarray setup. 

To ensure the comparability of the two fluorescent deoxynucleotides for distinct labeling, miRNA synthetic templates were labeled using corresponding miRNA-specific oligonucleotides in two different cZIP combinations. Signal intensities are regarded as comparable if signals can be detected for both labeling variants (signal to noise ratio > 3). Mature synthetic miRNAs were labeled with Cy3-dUTP and dTTP using MSO-cZIP set A; precursor synthetic miRNAs were reverse transcribed with Cy3-dATP and unlabeled deoxynucleotides using MSO-cZIP set B. 

**Figure 5:**
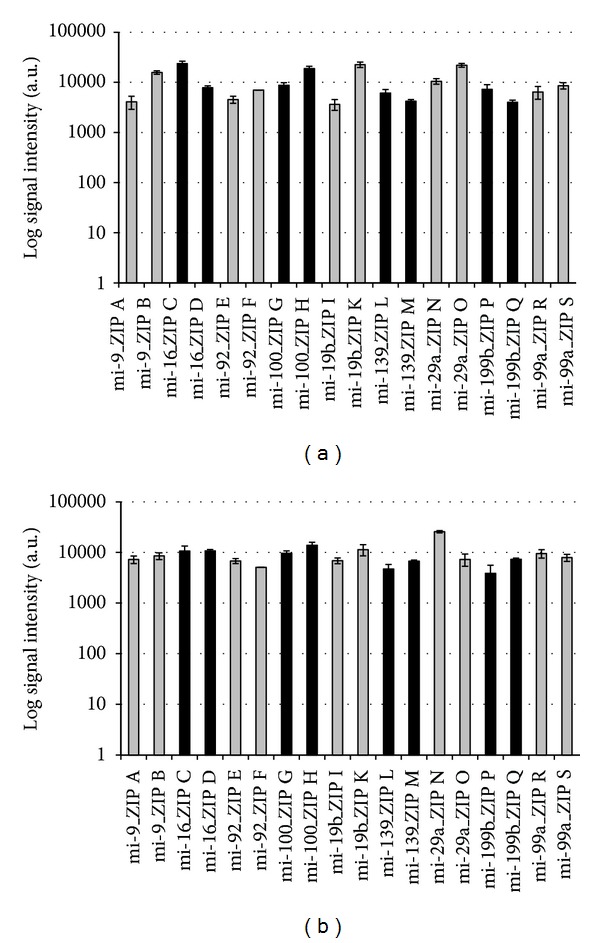
Comparison of mature and precursor miRNA labeling with different fluorescent deoxynucleotides. Log signal intensities from three hybridizations after labeling of mature synthetic miRNA with Cy3-dUTP and unlabeled dTTP in combination with MSO-cZIP set A (a) or MSO-cZIP set B (b), and precursor synthetic miRNA with Cy3-dATP and unlabeled deoxynucleotides in combination with MSO-cZIP set B (a) or MSO-cZIP set A (b) are shown. Comparative cZIPs for each miRNA are illustrated contiguous in identical colors.

Further, synthetic miRNA templates were labeled the other way round, resulting in mature synthetic miRNA labeled with MSO-cZIP set B and precursor synthetic miRNAs with MSO-cZIP set A, respectively. Comparable signal intensities for corresponding mature and precursor miRNA were detected for all sets (see also Figures 5(a) and 5(b), resp.). This allows the combination of both labelled deoxynucleotides for the simultaneous detection of mature and precursor miRNAs in one DNA microarray experiment. 

The detection range of the system was estimated using miRNA synthetic templates in a range of four orders of magnitude as input for different labeling reactions.

Mature miRNA synthetic templates were labeled with Cy3-dUTP and unlabeled dTTP using MSO-cZIP set A, whereas precursor miRNA synthetic templates were labeled with Cy3-dATP and unlabeled deoxynucleotides, using MSO-cZIP set B.

At first, mature miRNA synthetic templates were varied (10-fold dilution series with 0.3–0.003 pmoles), while the precursor miRNA synthetic template amount was kept constant at 3 pmoles. Experiments were performed in triplicates. 

**Figure 6:**
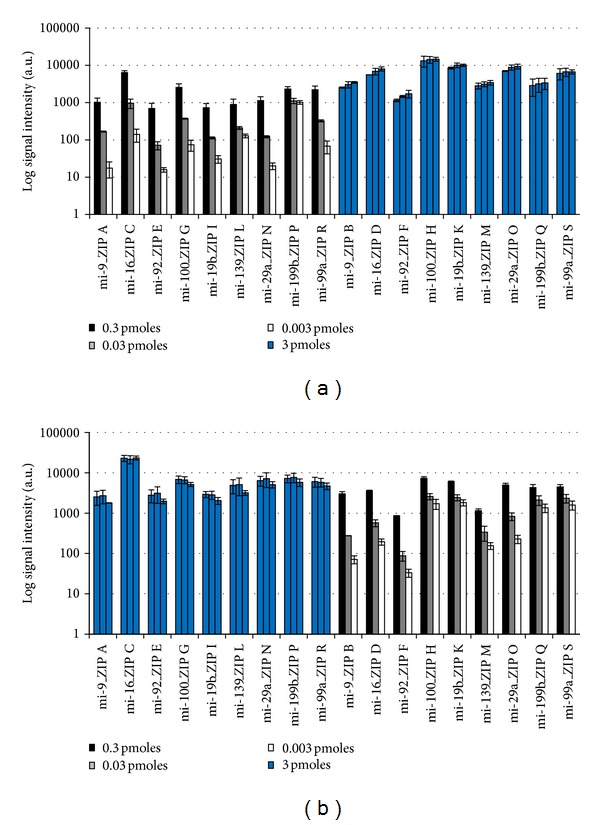
Simultaneous detection of mature and precursor miRNAs over four orders of magnitude. Log signal intensities from three hybridizations of precursor and mature miRNAs in different amounts are shown. (a) Mature miRNA synthetic templates variable (0.3, 0.03, 0.003 pmoles), precursor miRNA synthetic templates constant (3 pmoles each); (b) mature miRNA synthetic templates constant (3 pmoles each), precursor miRNA synthetic templates variable (0.3, 0.03, 0.003 pmoles). ZIPs for mature miRNA detection: A, C, E, G, I, L, N, P, and R. ZIPs for precursor miRNA detection: B, D, F, H, K, M, O, Q, and S.

For all ZIP-code miRNA combinations, a minimum detection range over 2-3 orders of magnitude could be shown (see Figure 6(a)). Five ZIP-code miRNA combinations (mi-16, mi-100, mi-139, mi-199b, mi-99a) exhibited even a detection range over three orders of magnitude from 0.03 up to 3 pmoles. 

In addition, mature miRNA synthetic template amount was kept constant (3 pmoles) in all three hybridizations, whereas precursor miRNA synthetic templates varied in 10-fold dilution steps from 0.3 down to 0.003 pmoles. A detection range of the system over three orders of magnitude was confirmed for 7 out of 9 tested ZIP-code pair-miRNA combinations (for mi-16, mi-100, mi-19b, mi-139, mi-29a, mi-199b, mi-99a) as shown in Figure 6(b). For mi-16 and mi-29a, a detection range over four orders of magnitude (from 0.003 up to 3 pmoles) was observed.

Altogether, a detection range at least over three orders of magnitude was achieved using probe sets mi-16, mi-100, mi-139, mi-29a, mi-199b, and mi-99a, so these probe sets were proven to be qualified for quantification. Regarding the probe sets for mi-9, mi-92, and mi-19b, a qualitative miRNA detection based on *on-off* signals is possible.

The experimental setup is based on the direct comparison of mature and precursor miRNA templates and might, therefore, possibly be influenced by template quantification biases. Nevertheless, we were able to show an average detection range of our system over three orders of magnitude for the majority of cases, for two miRNAs even up to four orders of magnitude.

**Figure 7:**
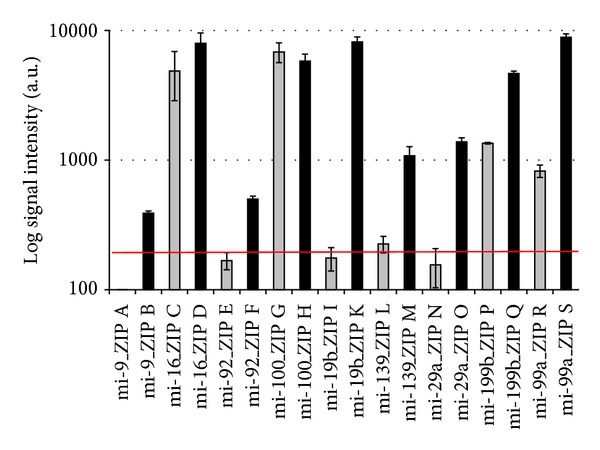
Detection of mature and precursor amiR in HeLa cells. Signal intensities from three hybridizations of fluorescently labeled MSO-cZIPs with the ZIP-code array after specific labeling are presented. Mature amiR was labeled with Cy3-dUTP and unlabeled dTTP using MSO-cZIP set A (gray), precursor amiR was labeled with Cy3-dATP and unlabeled deoxynucleotides using MSO-cZIP set B (black). ZIPs for mature miRNA detection: A, C, E, G, I, L, N, P and R. ZIPs for precursor miRNA detection: B, D, F, H, K, M, O, Q and S. The red line indicates the defined threshold value (190 a.u.).

After establishment of the method, the assay was applied to analyze human tumor cells. Both mature and precursor miRNAs amplified from HeLa cells were specifically labeled using the described method. Individual signals for mature and precursor amiRs were detected with the ZIP-code array (Table 4; Figure 7).

To confirm the findings from array-based mature and precursor amiR detection in HeLa cells, we used qRT-PCR as an independent method for validation. A set of four miRNAs was chosen, which passed our expected criteria from the establishment with synthetic miRNA templates above. qRT-PCR was used to check for *on-off* signals, that is, the presence or absence of corresponding mature and precursor miRNA forms.

qRT-PCR amplification curves were generated for the miRNA pairs miR-16 and pre-miR-16, miR-9 and pre-miR-9, miR-99a and pre-miR-99a, and miR-199b and pre-miR-199b (for PCR efficiencies please see Table 3). As expected, in melting curve analyses and 3% agarose gel electrophoresis, only the expected amplicon (either mature or precursor) for each primer pair could be detected (see Figure 1). Ct values from qRT-PCR are listed in Table 4.

In order to determine absence or presence of a particular miRNA in qRT-PCR assays, a threshold Ct value was defined based on quantification of genomic DNA from HaCaT cell line for pre-miR-16, pre-miR-99a, and pre-miR-199b in dilution steps from 2.4 × 10^5^–2.4 × 10^1^ copies/well. Copy numbers were calculated according to Lemuth et al. [41]. A copy number of 2.4 × 10^5^, 2.4 × 10^4^, 2.4 × 10^3^, and 2.4 × 10^2^ corresponded to average Ct values of 21.7 ± 1.7, 25.2 ± 2.2, 29.0 ± 2.0, and 34.65, respectively. According to this the sensitivity of the assays was set to 2.4 × 10^2^ copies corresponding to a Ct value of 34.65. Ct values equal or below this threshold indicate presence of pre-miR and miR (Table 4).

In accordance with DNA microarray signal intensities, the presence of miR-16, pre-miR-16, miR-99a, pre-miR-99a, miR-199b, and pre-miR-199b in HeLa cells was confirmed by qRT-PCR. 

Inconsistencies between our array and qPCR results were only detected for pre-miR-9 and miR-9. The array data indicate no presence of mature miR-9, whereas the qPCR indicates limited amounts of mature miR-9, although at levels close to the threshold. Comparing these data to data from the literature, two independent investigations using different methods show that mature miR is not expressed in HeLa cells [38, 39]. This indicates that the qRT-PCR data in this case might give a low but reproducible false-positive signal. For the precursor miR-9 no signal was detected in the qPCR, whereas the array data showed a low but reproducibly detectable signal for pre-miR-9. In this case, the PCR efficiency was the least efficient for all miR tested. Taking these results together into account, especially with regard to the literature data, indicates that most likely the qPCR for miR-9 and pre-miR-9 renders the critical results. Also the low signal intensities might further facilitate false-positive and false-negative results. qRT-PCR setup of miRNAs are very limited in placement of primers. This limitation often renders the design of accurate qPCR tools a challenging task.

The array results for all other tested mature miRNAs are in complete accordance with both our qPCR and the literature data (see Table 4). 

In summary, we have established a method for the simultaneous detection and differentiation of mature and precursor miRNA molecules based on a ZIP-code array system. 

Using synthetic miRNA templates, we clearly demonstrated the specificity of the method for the different miRNA types, as well as a detection range up to four orders of magnitude. Moreover, we applied our method to amiR from the cells. The detection of mature and precursor miRNAs was validated in a human tumor cell line model. 

The method presented has the ability to detect any known miRNA sequence. This is important as mature miRNA was found to be present in different length variants in cells [42–44]. These miRNA isoforms can alter miRNA target regulation [43], changing their role in tumorigenesis. Our method offers the possibility for the comprehensive detection of known miRNA variants, depending on the available number of ZIPs on the DNA microarray. 

The here studied miRNAs are located on separate pre-miRNA molecules. If the assay is to be transferred to polycistronic miRNAs, labeling of pre-miRs could be affected by sterical hindrance through miR-primer binding in multiplex miR labeling. This would have to be clarified in further studies.

Based on synthetic templates, we have proven the qualification of our method for the simultaneous detection and distinction of mature and precursor miRNAs in principle, and in order to further improve our method, an optimization of the MSO position could be pursued.

Regarding the detection of miRNA in cells, a further optimization could also be performed by the establishment of the ZIP-code system based on L-DNA analogue to Hauser et al. [35] with the aim of reducing background signals and preventing potential cross-hybridizations with D-DNA.

In conclusion, the comparative high-throughput-based detection of mature and precursor miRNA as tumor markers including variants has the potential for an improved tumor classification. This could allow an accurate diagnosis for a promising therapy, especially in the case of very heterogeneous tumor entities.

## Figures and Tables

**Table 1:** Sequences of mature and precursor synthetic miRNA templates. Synthetic miRNA templates composed of reverse complementary miRNA sequence (bold), 14-mer poly d(T) sequence (standard) and reverse complementary T7 RNA polymerase promoter sequence (italic). Synthetic templates of four precursor synthetic miRNAs were adapted to a maximum length of 80 bases*.

miRBase ID	miRBase accession number	Sequence (5′ to 3′ orientation)
hsa-miR-9-5p	MIMAT0000441	**TCA TAC AGC TAG ATA ACC AAA GA** TTTTTTTTTTTTTT *CCCTATAGTGAGTCGTATTA *
hsa-mir-9-3	MI0000468	**CA CTC ATA CAG CTA GAT AAC CAA AGA GAG AAA CGG GCCTCC** TTTTTTTTTTTTTT *CCCTATAGTGAGTCGTATTA *
hsa-miR-16-5p	MIMAT0000069	**CGC CAA TAT TTA CGT GCT GCT A** TTTTTTTTTTTTTT *CCCTATAGTGAGTCGTATTA *
hsa-mir-16-1	MI0000070	**TAA CGC CAA TAT TTA CGT GCT GCT AAG GCA CTG CTG AC** TTTTTTTTTTTTTT *CCCTATAGTGAGTCGTATTA *
hsa-miR-92a-3p	MIMAT0000092	**ACA GGC CGG GAC AAG TGC AAT A** TTTTTTTTTTTTTT *CCCTATAGTGAGTCGTATTA *
hsa-mir-92a-1*	MI0000093	**TCA ACA GGC CGG GAC AAG TGC AAT ACC ATA CAG AAA CA** TTTTTTTTTTTTTT *CCCTATAGTGAGTCGTATTA *
hsa-miR-100-5p	MIMAT0000098	**CAC AAG TTC GGA TCT ACG GGT T** TTTTTTTTTTTTTT *CCCTATAGTGAGTCGTATTA *
hsa-mir-100	MI0000102	**TA CCA CAA GTT CGG ATC TAC GGG TTT GTG GCA ACA GG** TTTTTTTTTTTTTT *CCCTATAGTGAGTCGTATTA *
hsa-miR-19b-3p	MIMAT0000074	**TCA GTT TTG CAT GGA TTT GCA CA** TTTTTTTTTTTTTT *CCCTATAGTGAGTCGTATTA *
hsa-mir-19b-1*	MI0000074	**C AGT CAG TTT TGC ATG GAT TTG CAC AGC AGA ATA TCA C** TTTTTTTTTTTTTT *CCCTATAGTGAGTCGTATTA *
hsa-miR-139-5p	MIMAT0000250	**CTG GAG ACA CGT GCA CTG TAG A** TTTTTTTTTTTTTT *CCCTATAGTGAGTCGTATTA *
hsa-mir-139	MI0000261	**AC ACT GGA GAC ACG TGC ACT GTA GAA TAC AC** TTTTTTTTTTTTTT *CCCTATAGTGAGTCGTATTA *
hsa-miR-29a-3p	MIMAT0000086	**TAA CCG ATT TCA GAT GGT GCT A** TTTTTTTTTTTTTT *CCCTATAGTGAGTCGTATTA *
hsa-mir-29a*	MI0000087	**ATA ACC GAT TTC AGA TGG TGC TAG AAA ATT ATA TTG AC** TTTTTTTTTTTTTT *CCCTATAGTGAGTCGTATTA *
hsa-miR-199b-5p	MIMAT0000263	**GAA CAG ATA GTC TAA ACA CTG GG** TTTTTTTTTTTTTT *CCCTATAGTGAGTCGTATTA *
hsa-mir-199b*	MI0000282	**C CTG AAC AGA TAG TCT AAA CAC TGG GTA GAC GGA GTG GAG** TTTTTTTTTTTTTT *CCCTATAGTGAGTCGTATTA *
hsa-miR-99a-5p	MIMAT0000097	**CAC AAG ATC GGA TCT ACG GGT T** TTTTTTTTTTTTTT *CCCTATAGTGAGTCGTATTA *
hsa-mir-99a	MI0000101	**C ACC ACA AGA TCG GAT CTA CGG GTT TAT GCC AAT GGG** TTTTTTTTTTTTTT *CCCTATAGTGAGTCGTATTA *

**Table 2:** Sequences of ZIP oligonucleotides and miRNA-specific oligonucleotides-cZIP. Sequences of ZIP oligonucleotides (immobilized on DNA microarray) and miRNA-specific oligonucleotides with cZIP (miRNA-specific sequences are illustrated in bold).

Internal ZIP ID	MSO-cZIP set	Sequence of ZIP oligonucleotide on DNA microarray (5′ to 3′ orientation)	Sequence of miRNA-specific oligonucleotide-cZIP (MSO-cZIP) (5′ to 3′ orientation)
mi-9_ZIP A	A	GCAGTGCTCACCGTCCGCGA	TCGCGGACGGTGAGCACTGC **TCATACAGCTAGATAACCAAAGA**
mi-9_ZIP B	B	ACGCGACGCACCTGCTCCAA	TTGGAGCAGGTGCGTCGCGT **TCATACAGCTAGATAACCAAAGA**
mi-16_ZIP C	A	CGGAGTGGCACCAGCGGGAA	TTCCCGCTGGTGCCACTCCG **CGCCAATATTTACGTGCTGCTA**
mi-16_ZIP D	B	GTGGGAGCGACGCAGGGCAG	CTGCCCTGCGTCGCTCCCAC **CGCCAATATTTACGTGCTGCTA**
mi-92_ZIP E	A	TGGCGGTCTGCTGAGCGGTC	GACCGCTCAGCAGACCGCCA **ACAGGCCGGGACAAGTGCAATA**
mi-92_ZIP F	B	GCCTCGAGCCAACACCGCCT	AGGCGGTGTTGGCTCGAGGC **ACAGGCCGGGACAAGTGCAATA**
mi-100_ZIP G	A	TGGCCGGACAGGAGACACGC	GCGTGTCTCCTGTCCGGCCA **CACAAGTTCGGATCTACGGGTT**
mi-100_ZIP H	B	GCCTGCCTTCACGAGCCCAA	TTGGGCTCGTGAAGGCAGGC **CACAAGTTCGGATCTACGGGTT**
mi-19b_ZIP I	A	TGGCCGAGACTGCAGGAGCG	CGCTCCTGCAGTCTCGGCCA **TCAGTTTTGCATGGATTTGCACA**
mi-19b_ZIP K	B	ACGCCCTCCCAACCTCACGC	GCGTGAGGTTGGGAGGGCGT **TCAGTTTTGCATGGATTTGCACA**
mi-139_ZIP L	A	AGCGGACGACTGCGGACGAG	CTCGTCCGCAGTCGTCCGCT **CTGGAGACACGTGCACTGTAGA**
mi-139_ZIP M	B	TGGCGAGCGCAGTGGCAGAC	GTCTGCCACTGCGCTCGCCA **CTGGAGACACGTGCACTGTAGA**
mi-29a_ZIP N	A	AGCGTGCTGGTCGTGGGCCT	AGGCCCACGACCAGCACGCT **TAACCGATTTCAGATGGTGCTA**
mi-29a_ZIP O	B	AGCGCCAAGGGTCCTCGGGT	ACCCGAGGACCCTTGGCGCT **TAACCGATTTCAGATGGTGCTA**
mi-199b_ZIP P	A	GGGTGGTCCGGAGCGAGCAG	CTGCTCGCTCCGGACCACCC **GAACAGATAGTCTAAACACTGGG**
mi-199b_ZIP Q	B	CCTCAGACGGGTAGCGGCGA	TCGCCGCTACCCGTCTGAGG **GAACAGATAGTCTAAACACTGGG**
mi-99a_ZIP R	A	CCTCGTCCGTCCGGTCACCA	TGGTGACCGGACGGACGAGG **CACAAGATCGGATCTACGGGTT**
mi-99a_ZIP S	B	GGGTGTGGACGCGGAAGCAG	CTGCTTCCGCGTCCACACCC **CACAAGATCGGATCTACGGGTT**

**Table 3:** miRNA-specific primers and assays used in quantitative real-time PCR. miRNA-specific commercial assays (Qiagen) and sequences of self-made miRNA-specific primers used in qRT-PCR (Tm = melting temperature).

miRBase ID	MiScript Assay (Qiagen)	Forward primer/reverse primer (5′ to 3′ orientation)	Length (nt)	Tm (°C)	Amplicon length (bp)	PCR efficiency	Error rate (%)
hsa-miR-9-5p	Hs_miR-9_1 miScript Primer Assay				85–87	2.3	4.90
hsa-mir-9-3		GAGGCCCGTTTCTCTCTTTGG/	21	59	63	1.6	3.91
		CTAGCTTTATGACGGCTCTGTGG	23	59			
hsa-miR-16-5p	Hs_miR-16_2 miScript Primer Assay				85–87	1.9	2.11
hsa-mir-16-1		GCAGCACGTAAATATTGGCGTTAAG/	25	59	74	1.8	0.61
		GTCAACCTTACTTCAGCAGCACAG	24	60			
hsa-miR-199b-5p	Hs_miR-199b_1 miScript Primer Assay				85–87	2.5	3.47
hsa-mir-199b	Hs_mir-199b_1_PR miScript Precursor Assay				89	1.9	0.45
hsa-miR-99a-5p	Hs_miR-99a_2 miScript Primer Assay				85–87	1.9	1.34
hsa-mir-99a	Hs_mir-99a_1_PR miScript Precursor Assay				72	1.9	4.15

**Table 4:** Validation of DNA microarray-based miRNA detection in human cells. Mature and precursor miRNA detection in HeLa cells by DNA microarray, validation of array signals by comparison to qRT-PCR data and/or comparison to literature data for mature miRNAs. The presence of individual miRNAs (Figure 7) was determined based on array signal intensity (threshold 190 a.u., according to Figure 3(a)) or qRT-PCR Ct values equal or below a threshold Ct value of 34.65 (corresponding to 2.4 × 10^2^ copies).

miRBase ID	Internal ZIP ID	Mature/precursor miRNA	Presence of miRNA according to DNA microarray results	Presence of miRNA according to qRT-PCR (average Ct value from triplicates)	Presence of miRNA according to Reference
hsa-miR-9-5p	mi-9_ZIP A	Mature	No	Yes (33.1)	No [38, 39]
hsa-mir-9-3	mi-9_ZIP B	Precursor	Yes	No (>40.0)	
hsa-miR-16-5p	mi-16_ZIP C	Mature	Yes	Yes (18.1)	
hsa-mir-16-1	mi-16_ZIP D	Precursor	Yes	Yes (34.6)	
hsa-miR-92a-3p	mi-92_ZIP E	Mature	No	n.d.	No [39]
hsa-mir-92a-1	mi-92_ZIP F	Precursor	Yes	n.d.	n/a
hsa-miR-100-5p	mi-100_ZIP G	Mature	Yes	n.d.	Yes [40]
hsa-mir-100	mi-100_ZIP H	Precursor	Yes	n.d.	n/a
hsa-miR-19b-3p	mi-19b_ZIP I	Mature	No	n.d.	No [39, 40]
hsa-mir-19b-1	mi-19b_ZIP K	Precursor	Yes	n.d.	n/a
hsa-miR-139-5p	mi-139_ZIP L	Mature	Yes	n.d.	Yes [40]
hsa-mir-139	mi-139_ZIP M	Precursor	Yes	n.d.	n/a
hsa-miR-29a-3p	mi-29a_ZIP N	Mature	No	n.d.	No [39, 40]
hsa-mir-29a	mi-29a_ZIP O	Precursor	Yes	n.d.	n/a
hsa-miR-199b-5p	mi-199b_ZIP P	Mature	Yes	Yes (31.4)	
hsa-mir-199b	mi-199b_ZIP Q	Precursor	Yes	Yes (32.0)	
hsa-miR-99a-5p	mi-99a_ZIP R	Mature	Yes	Yes (21.1)	
hsa-mir-99a	mi-99a_ZIP S	Precursor	Yes	Yes (33.4)	
